# AURORA: Bariatric surgery registration in women of reproductive age - a multicenter prospective cohort study

**DOI:** 10.1186/2049-3258-73-S1-P45

**Published:** 2015-09-17

**Authors:** Goele Jans, Christophe Matthys, Matthias Lannoo, Bart Van der Schueren, Bruno Dillemans, Luc Lemmens, Jean-Pierre Saey, Yves Van Nieuwenhove, Ben De Becker, Pascale Grandjean, Hilde Logghe, Kristien Roelens, Anne Loccufier, Johan Verhaeghe, Roland Devlieger

**Affiliations:** 1 KU Leuven, Leuven, Belgium; 2 KU Leuven/University of Leuven, Leuven, Belgium; 3 AZ St-Jan, Bruges, Belgium; 4 AZ St-Nikolaas, St-Niklaas, Belgium; 5 CHR Mons-Hainaut, Mons-Hainaut, Belgium; 6 UZ Gent, Gent, Belgium; 7 AZ St-Augustinus, Wilrijk, Belgium; 8 AZ St-Lucas, Bruges, Belgium

## Introduction

Bariatric surgery has become the most successful long-term treatment of severe obesity, illustrated by almost 469,000 performed procedures worldwide in 2013. Women aged 18-45 years represent the major part of the bariatric population. A long-term follow-up, starting before surgery until a subsequent pregnancy and postpartum period, is needed to study reproduction outcomes in order to fill knowledge gaps and provide a scientific base for clinical guidelines.

## Methods/design

This multicenter prospective cohort study aims to examine the impact of bariatric surgery on reproduction outcomes. Women aged 18-45 years are invited to participate at 4 possible inclusion moments: 1) pre-operatively, 2) post-operatively, 3) before 15 weeks of pregnancy and 4) in the early postpartum period. Depending on the time of inclusion, data is collected pre-operatively, 3 weeks and 3, 6 and 12 months post-operatively and during the first, second and third trimester of pregnancy, at delivery, weekly until 6 weeks postpartum and finally at 6 months postpartum. Online questionnaires (contraceptive use, sexuality, menstrual cycle, intention of becoming pregnant, diet, physical activity, psycho-social health and nutritional supplement intake) are distributed on the different measuring moments. Fasting blood samples are collected to determine the nutritional status, measuring vitamin A, D, E, K, B-1, B-12 and folate, albumin, total protein, hemoglobin, hematocrit, iron, ferritin, transferrin (saturation), prothrombin time, activated partial prothrombin time, magnesium, calcium and zinc. Participants will be weighted every measurement point. Ultrasounds and pregnancy outcomes are reported every trimester of pregnancy. Breast milk samples of 5 ml are collected weekly from after birth until the routine 6 week postpartum consultation in order to examine the milk macronutrient composition.

## Conclusion

AURORA is a unique multicenter cohort study and might be of great value for the development of guidelines and recommendations in order to optimize clinical care in these women.

